# A silent epidemic: Exploring the clinico-epidemiological impact of explosion and gunshot injuries in the emergency department of a tertiary hospital in Somalia

**DOI:** 10.1016/j.afjem.2025.100898

**Published:** 2025-08-29

**Authors:** Hussein Hassan Mohamed, Hassan Adan Ali Adan, Selim Turfan, Murat Aysin, Mohamed Farah Yusuf Mohamud

**Affiliations:** a Emergency Department, Mogadishu Somali Turkey Training and Research Hospital, Mogadishu, Somalia; b BalikesirUniversity Faculty of Medicine, Department of Public Health, Balikesir, Turkey; c Facult of Medicine and Surgery, Mogadishu University, Mogadishu, Somalia; d Tayo Institute for Research and Development, Mogadishu, Somalia

**Keywords:** Mass casualty, Gunshot wounds, Explosive injuries, Trauma care, Triage

## Abstract

**Background:**

Mass casualty incidents, such as explosions and gunshot wounds (GSWs), pose significant public health challenges. This study analyzes the clinico-epidemiological profile and outcomes of patients with explosive injuries and GSWs in Somalia.

**Method:**

A retrospective analysis was conducted on 225 patients admitted to the Emergency Department of a tertiary hospital in Somalia between January and December 2021. Data collected included injury type, anatomical distribution, demographics, hospital admissions, and outcomes.

**Results:**

Of the 225 explosion and GSW injuries, explosive injuries accounted for 58 %, while GSWs made up 42 %. The majority of patients were male (85.3 %), with 89.5 % in the GSW group and 82.3 % in the explosion group. More than half(58.7 %) of the patients were aged 18 to 30 years, with 59.2 % in the explosion group and 57.9 % in the GSW group. Anatomical analysis revealed that head injuries were most common(21 %), particularly in explosion cases, as well as higher rates of head (26.2 %), maxillofacial(7.7 %), and lower-limb injuries (12.3 %) compared to GSW patients. Some 21.7 % of patients were discharged from ED, 19 % admitted to ICU and an inpatient death rate of 12.9 %, including three patients (1.3 %) who died in the Emergency Department, all from the explosion injury group.

**Conclusion:**

The rising incidence of traumatic injuries necessitates a multifaceted approach, including enhanced emergency response systems and public health initiatives. This data serves as a call to action for healthcare providers and policymakers to prioritize the management and prevention of explosion and gunshot-related injuries in Somalia.

## African relevance

• High rates of explosion and gunshot injuries in Somalia highlight a significant public health issue in conflict-affected African regions.
• The study highlights the challenges Emergency Departments encounter in managing mass casualties, underscoring the need for enhanced trauma care protocols.
• The findings advocate for evidence-based policies such as the establishment of National Trauma Protocols and an Emergency Medical Service (EMS) System, training programs for emergency care providers, community awareness campaigns, and partnerships with local and international NGOs to improve patient outcomes in similar African contexts.

## Introduction

Globally, there is an increase in mass casualty incidents (MCIs) and disasters that cause significant mortality and morbidity [1,2]. These incidents can be attributed to two main factors: natural disasters such as floods, cyclones, and earthquakes, as well as man-made causes including industrial disasters, road traffic accidents (RTAs), gender-based violence (GBV), terrorist-related explosions, and armed conflicts [1]. Trauma victims in low- and middle-income countries (LMIC) experience higher rates of morbidity and mortality compared to those in high-income countries [3,4]. This highlights the need for improved trauma surveillance and care as an effective strategy to reduce global health disparities. Although the impact of trauma is widely acknowledged, few studies describe its prevalence and causes in developing regions [3,5].

During the past two decades, terrorist attacks and armed conflicts have resulted in the loss of over two million lives and more than 1.4 million lives [6]. The countries most significantly impacted by this significant fatality rate include Somalia, Afghanistan, Ethiopia, Bangladesh, the Congo Republic, Indonesia, Iraq, Liberia, Pakistan, and Sudan. Explosions and fires from various sources account for over 70 % of disasters, resulting in more than 20 % of fatalities at the incident scene [7]. Whether caused by accident or intention, explosive events can lead to significant injuries, contributing to heightened morbidity and mortality rates [8]. Moreover, the extensive use of Improvised Explosive Devices has led to the emergence of new injury patterns characterized by severe wounds [9]. Since World War II, approximately 70 % of injuries sustained in conflicts have been due to explosions; however, during the initial stages of conflicts, gunshot injuries are more prevalent [10]. Additionally, gunshot injuries are three times more common in developing and low-resource countries than in developed nations [11].

For over three decades, Somalia has experienced a multifaceted humanitarian crisis marked by the collapse of its health infrastructure, prolonged warfare, and terror-related injuries, resulting in thousands of deaths and widespread disabilities [12,13]. This study was conducted to describe the epidemiological characteristics and outcomes of terrorist attacks presenting to the emergency department and highlight their frequency, mechanisms, injury patterns, severity, and outcomes.

## Method

### Study design and setting

A retrospective, hospital-based, cross-sectional study was conducted from January to December 2021 at the Mogadishu-Somali-Turkey Training and Research Hospital in Mogadishu, Somalia, to review the clinico-epidemiological profile and case outcomes of gunshot and explosive injuries.

### Study population and setting

The authors systematically reviewed the records of all patients who presented to the emergency department of Mogadishu Somali Turkey Training and Research Hospital (MSTTRH), Mogadishu, Somalia, with injuries from bombing/explosion and gunshot attacks during the study period. Data was retrieved from the hospital's medical database (FONET). FONET is an electronic medical record (EMR) system that allows access to personal and clinical patient records.

MSTTRH is the sole teaching and referral trauma and emergency hospital in the country, catering to a population exceeding 5 million. It is well-known for its extensive emergency care services, making it a key facility to analyze clinical trends and outcomes related to traumatic injuries in the area. The hospital employs around 1000 staff members, with over 600 in medical roles. It features more than 250 inpatient beds, 30 adult ICU beds, 10 pediatric ICU beds, 25 neonatal ICU beds, and offers comprehensive specialist services through over 15 operational departments, including an. Emergency Department offering comprehensive 24-hour emergency care [[14], [15], [16]].

Prehospital care and patient transport are severely limited in Somalia. There is no established emergency medical service (EMS) system; only a few governmental, NGO, and private ambulances involved in patient transport. These ambulances often lack paramedics, resulting in a total absence of prehospital care. Additionally, challenges such as insufficient training for healthcare personnel, a shortage of medical supplies, and inadequate infrastructure further hinder effective emergency response and patient care. In addition to these logistical challenges, the public's awareness of emergency services is limited. Many people may not know how to access available services or may hesitate to call for help due to a lack of trust in the system. This can lead to delays in treatment that are crucial for survival in emergency situations.

### Inclusion and exclusion criteria

All patients who sustained firearm and explosive injuries and were brought to the Emergency Eepartment were included in the study. Excluded from the study were victims with incomplete data and patients who were deemed dead at the scene or upon arrival at the hospital, as those who were dead on arrival did not receive any prehospital care, making it challenging to assess the impact of emergency interventions. While mortality data can provide valuable insights into the severity and intent of the injuries, the focus of this study was on the clinical outcomes and management of those who received care. Patients with injuries due to traffic accidents, falls, GSWs not linked to terrorism, and violence or assaults were also excluded from the study.

### Ethical consideration

Ethical approval was obtained from the local ethical committee board of the Mogadishu, Somali Turkish Training and Research Hospital with reference number MSTH/9568. The institution's Ethical Committee waived the need for informed consent due to the use of electronic medical records and the absence of harm to patients, ensuring that ethical standards were upheld throughout the study.

### Data collection

Data were collected from the hospital information management system (HIMS) database (FONET) and included information such as sex, age, cause of trauma, types of injuries, location and severity of injuries, definitive diagnosis, site of admission, and patient outcomes. Terrorism-related injuries were identified using specific ICD codes: Y24, which refers to *Injuries caused by explosives*, and X96, which pertains to *Assault by firearm*, including injuries from gunshot wounds related to terrorist acts. The data retrieval was conducted by two emergency medicine specialists who were familiar with the hospital's electronic medical record (EMR) system, ensuring accuracy and confidentiality.

### Data analysis

Statistical analysis was performed using the Statistical Package for the Social Sciences (SPSS) software v.26. Patients were categorized into two groups: those injured by explosions and those injured by gunshots. This analysis specifically pertains to injuries related to terrorist attacks, assuming that all GSWs and patients with explosive injuries presenting to the Emergency Department are casualties of such attacks. Frequencies and percentages were used to represent categorical variables. This was depicted by tables and charts to illustrate the characteristics of participants, frequency, mechanisms, injury patterns, severity, and outcomes.

## Results

During the study period, a total of 225 patients were recorded as sustaining injuries caused by terrorist acts. The majority of patients were injured by an explosion (*n* = 130, 58 %), while forty-two percent (95 patients) had gunshot wounds (GSW).

In terms of sex distribution, most of the participants (85.3 %, 192) were male, with the majority of male casualties resulting from explosive injuries, which accounted for 55.7 % (107 cases) of the total cases, as demonstrated in Table 1.

**Table 1:** Patient characteristics, injury severity score, and anatomical distribution of gunshot injured versus explosion injured groups**.**

Variables		Total (225), n ( %)	Mechanism of Injury		p Value
			Gunshot (95) n, %	Explosion (130) n, %	
**Sex**	Male	192 (85.3)	85 (89.5)	107 (82.3)	<0.001
	Female	33 (14.7)	10 (10.5)	23 (17.7)	
**Age Groups**	0–18	32 (14.2)	9 (9.5)	23 (17.7)	0.003
	19–30	132 (58.7)	55 (57.9)	77 (59.2)	
	31–50	53 (23.6)	28 (29.5)	25 (19.2)	
	≥51	8 (3.6)	3 (3.2)	5 (3.8)	
**ISS**	1–8	127 (56.4)	49 (51.6)	78 (60.0)	0.061
	9–15	48 (21.3)	27 (28.4)	21 (16.2)	
	16–24	24 (10,^7^)	9 (9.5)	15 (11.5)	
	25–75	26 (11.6)	10 (10.5)	16 (12.3)	
**Anatomical distribution**	Head injury	47 (20.9)	13 (13.7)	34 (26.2)	0.149
	Maxillofacial injury	17 (7.6)	7 (7.4)	10 (7.7)	
	Chest injury	27 (12.0)	19 (20.0)	8 (6.2)	
	Abdominal injury	25 (11.1)	11 (11.6)	14 (10.8)	
	Spinal and pelvic injury	10 (4.4)	5 (5.3)	5 (3.8)	
	Upper limb injury	15 (6.7)	8 (8.4)	7 (5.4)	
	Lower limb injury	24 (10.7)	8 (8.4)	16 (12.3)	
	Vascular Injury	12 (5.3)	6 (6.3)	6 (4.6)	

ISS; injury severity score.

More than half (132, 58.7 %) of the patients were between 19–30 years of age, 57.9 % in the GSW group and 59.2 % in the explosion group (Table 1). Patients injured in gunshot and explosion had a higher proportion of children (<19 years) when compared to the elderly (>50 years). Additionally, the youngest group (0–18 years) exhibited a notable difference, with a higher proportion of explosion injury patients (17.7 %) compared to gunshot injury patients (9.5 %).

The Injury Severity Score (ISS) revealed that explosive injuries are associated with a higher prevalence of severe and critical injuries (ISS > 16), affecting approximately 24 % of patients, compared to the gunshot victims experienced 20 %. However, patients injured by an explosion have a larger proportion of minor injuries and a larger proportion of critical to fatal injuries.

According to the anatomical distribution of injuries, the most frequently affected areas were as follows: the head, with a prevalence of 21 %; the extremities, with 17.4 %; the thorax, with 12 %; and the abdomen, with 11.1 %. Head, maxillofacial, and lower limb injuries were notably more prevalent among explosion injury patients (26.2 %, 7.7 %, and 12.3 %, respectively) compared to gunshot injury patients (13.7 %, 7.4 %, and 8.4 %). In contrast, the incidence of chest, abdomen, spinal, pelvic, upper limb, and vascular injuries was comparable across both groups, with gunshot injury patients experiencing a higher incidence compared to those with explosive injuries.

**Fig. 1:**
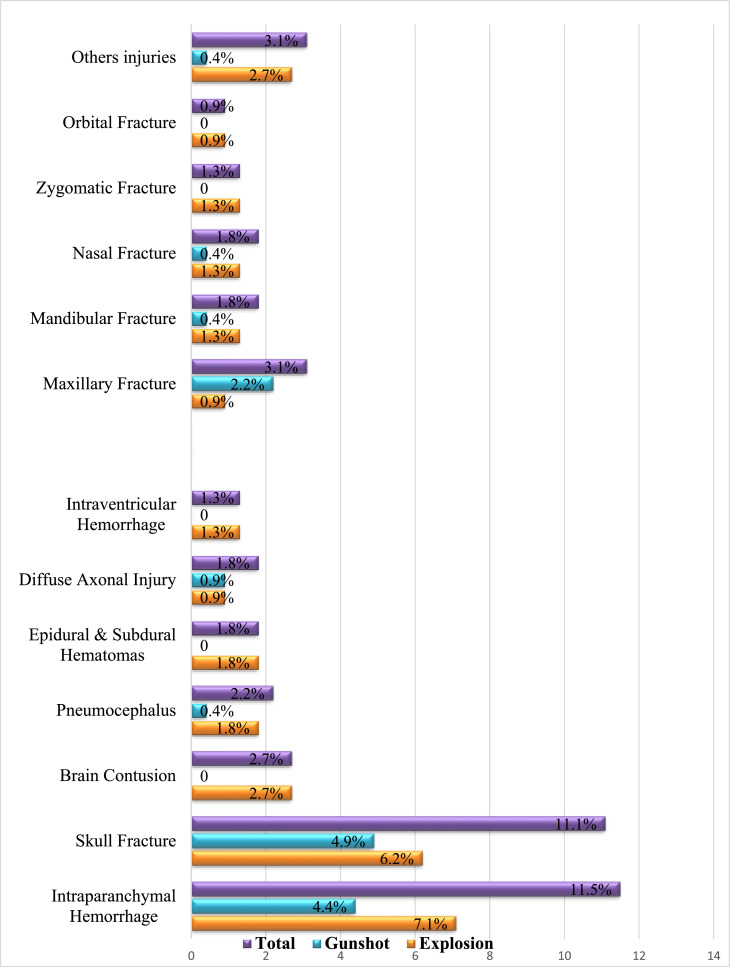
Pattern of Head and Maxillofacial Injuries.

The analysis of head injury patterns shown in Fig. 1 indicates that intracranial hemorrhage and skull fractures were the most common findings, comprising 11.5 % and 11.1 % of the total cases, respectively. Additionally, brain contusions, pneumocephalus, and subdural/epidural hematomas were reported in 2.7 %, 2.2 %, and 1.8 % of the cases, respectively. All of these injuries were more prevalent in explosive injuries compared to gunshot injuries, except for subdural/epidural hematomas, which were more common in gunshot injuries.

**Fig. 2:**
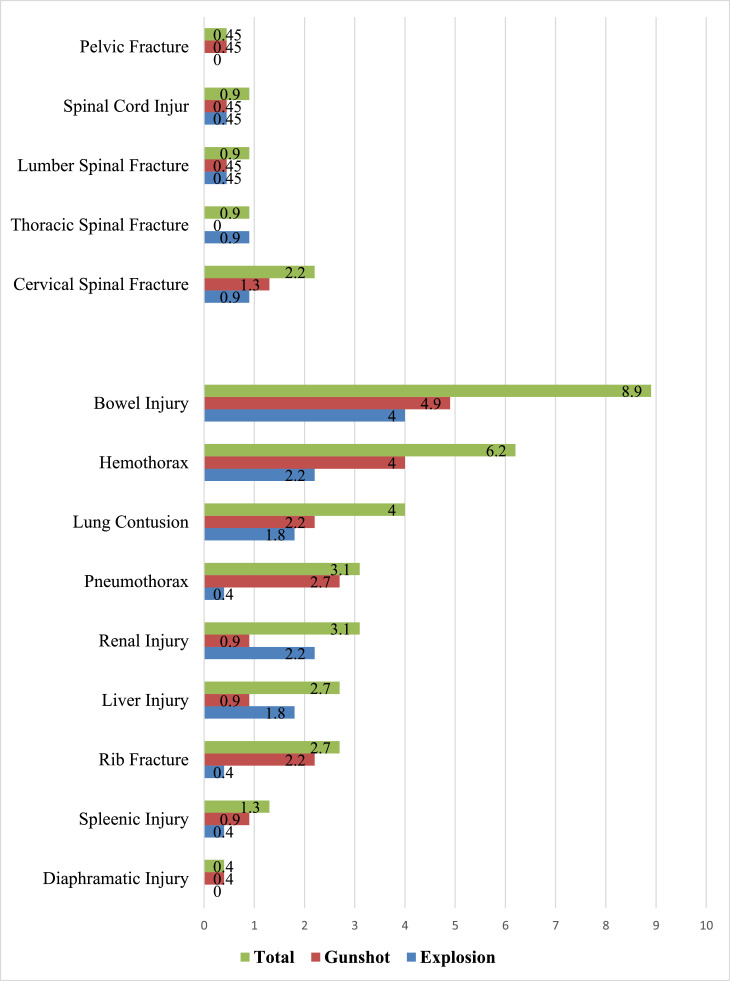
Pattern of Abdomino-thoratic, Spinal, and Pelvic injuries.

Maxillary fractures were the most common maxillofacial injuries. The abdomino-thoracic injury patterns presented in Fig. 2 indicate that bowel injuries were the most prevalent, affecting 8.9 % of the total cases. Hemothorax was present in 6.2 % of the total cases, while pneumothorax was reported in 3.1 % of the total cases. Liver injuries and rib fractures were also commonly observed, occurring in 2.7 % of the total cases for each. Notably, explosion-related incidents accounted for a significantly higher incidence of bowel injuries, pneumothorax, hemothorax, and rib fractures compared to gunshot wounds.

In terms of spinal and pelvic injuries, cervical spinal fractures were the most common, occurring in 5 cases, which accounted for 2.2 % of the total injuries. Cervical spinal fractures were more prominent in patients injured by gunshots compared to those injured by explosions, while thoracic spinal fractures were more prevalent in the explosion injury group.

**Fig. 3:**
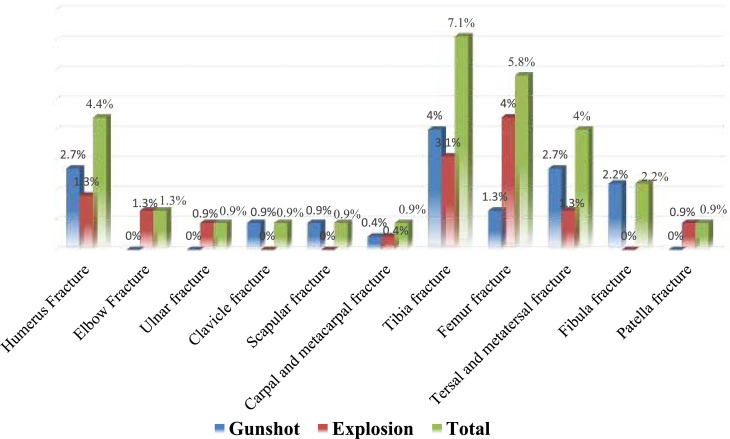
Pattern of upper and lower extremity injuries.

Examining the upper and lower extremity injuries, the data show that humerus fractures were the most prevalent in the upper extremity (4.4 %), followed by elbow fractures (1.3 %). In the lower extremity, tibia fractures were the most common (7.1 %), particularly in the explosion group, followed by femur, tarsal, and metatarsal, and fibula fractures (Fig. 3).

**Fig. 4:**
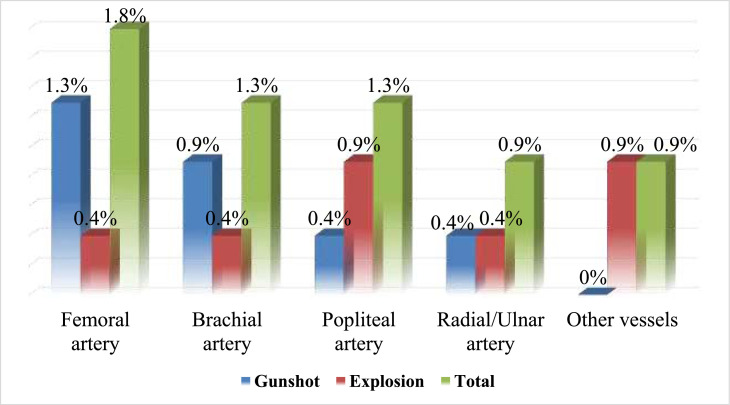
Pattern of vascular injuries.

Regarding vascular injuries, the brachial artery was the most frequently affected, accounting for 1.8 % of the total injuries, primarily in the explosion group. Popliteal artery injuries and radial/ulnar artery injuries were also reported, each with an incidence of 1.3 %, showing a relatively even distribution between the explosion and gunshot groups (Fig. 4).

Of the total patients, 49 (21.7 %) were discharged from the ED, with a significant difference in discharge rates between the two groups: 11.5 % of gunshot injury patients versus 29.2 % of explosion injury patients. Nearly half of the patients (105, 45.3 %) were admitted to various medical wards, with gunshot injury patients making up a higher percentage (59 %) compared to explosion injury patients (37.7 %). Notably, 43 patients (19.1 %) were admitted to the Intensive Care Unit (ICU), with similar rates observed for both types of injuries (19 % for gunshot and 19.2 % for patients with explosive injuries). Twenty patients (8.9 %) were taken directly to the operating theater, with a slightly higher proportion of explosion injury patients (10 %) compared to gunshot injury patients (7.3 %). Additionally, five patients (2.2 %) self-discharged from the hospital, with a higher percentage of gunshot injury patients (3.2 %) compared to explosion injury patients (1.5 %), indicating some patients felt well enough to leave, possibly reflecting less severe cases. Unfortunately, three patients (1.3 %) were recorded as dead in the emergency room, all of whom were from the explosion injury group, as shown in Table 2.

**Table 2:** Patient deposition and outcome.

Variables			Total, n ( %)	Mechanism of Injury		P Value
				Gunshot n, %	Explosion n, %	
**Patient Dsposition**	ED Discharge		49 (21.7)	11 (11.5)	38 (29.2)	<0.001*
	Medical ward		105 (45.3)	56 (59)	49 (37.7)	
	Intensive care unit		43 (19.1)	18 (19)	25 (19.2)	
	Direct to operating theater	Total	20 (8.9)	7 (7.3)	13 (10)	
		Limb injuries	6 (30)	2 (28.6)	4 (30.7)	
		Abdominal injuries	5 (25)	2 (28.6)	3 (23)	
		Vascular injuries	3 (15)	2 (28.6)	1 (7.7)	
		Head injuries	3 (15)	1 (14.2)	2 (15.4)	
		Thoratic injuries	2 (10)	0 (0)	2 (15.4)	
		Maxillofacial injuries	1 (5)	0 (0)	1 (7.7)	
	ED Self discharge		5 (2.2)	3 (3.2)	2 (1.5)	
	Died in the ED		3 (1.3)	0	3 (2.3)	
**Admission Departments**	Orthopedic		52 (23.1)	28 (29.5)	24 (18.5)	0.177
	Neurosurgery		40 (17.8)	14 (14.7)	26 (20.0)	
	General surgery		27 (12.0)	14 (14.7)	13 (10.0)	
	Thoraticsurgery		25 (11.1)	17 (17.9)	8 (6.2)	
	Otolaryngology		19 (8.4)	9 (9.5)	10 (7.7)	
	Cardiovascular Surgery		10 (4.4)	4 (4.2)	6 (4.6)	
	Opthalmology		1 (0.4)	0	1 (0.8)	
	Urology		1 (0.4)	1 (1.1)	0	
**Outcome**	Survived		196 (87.1)	84 (88.4)	112 (86.2)	0.031
	Hospital mortality		29 (12.9)	11 (11.6)	18 (13.8)	

ED; Emergency department.

Of the 225 patients, 196 (87.1 %) survived their injuries, with gunshot injury patients having a slightly higher survival rate of 88.4 % compared to 86.2 % for explosion injury patients. However, 29 patients (12.9 %) died during their hospital stay. The higher mortality rate among explosion injury patients highlights the severe nature of these injuries and the complexities involved in their treatment.

## Discussion

Mass casualty incidents, such as explosions and gunshot injuries, are significant public health concerns, especially in violent urban regions and conflict zones, necessitating a rapid, synchronized, and coordinated response from the emergency department team and other stakeholders to ensure optimal care delivery [2]. These types of injuries present unique challenges for medical professionals, requiring specialized management and treatment approaches to address the different mechanisms of trauma and its associated effects.

In this study, explosive injuries represented 58 % of the cases, while GSW made up 42 %. This distribution aligns with a study from Israel, which reported a total of 1155 terror-related injuries: 54 % by explosion, 36 % GSW, and 10 % by other means [17]. In a contrasting study from Somalia, it was found that most patients sustained injuries from gunshot wounds (66.7 %) rather than from explosions (33.3 %), based on an analysis of data from all patients injured in explosions and firearm attacks [18].

According to this analysis, in terms of sex distribution, the majority of participants were male, with explosive injuries accounting for a significant portion of the total male casualties. More than half of the patients were aged between 19–30 years, with a higher percentage in the gunshot wound group and a similar figure in the explosion group. Similar studies reported a high male predominance and that patients aged between 15–29 years made up a considerable portion, with explosive injuries being more prevalent [17,19].

Patients injured in gunshot and explosion had a higher proportion of children (<19 years) (14.2 %) when compared to the elderly (>50 years) (3.6 %). Additionally, the youngest group exhibited a notable difference, with a higher proportion of explosion injury patients compared to gunshot injury patients. A study conducted in Sudan reported that a significant percentage of the children in the study population were injured by gunshot wounds, making this age group the second most affected, following those aged 20 to 29 [20]. Another study from Jos University Teaching Hospital in North Central Nigeria revealed that out of 242 patients with gunshot injuries, 30 were children [21].

The anatomical distribution of injuries in this study revealed that the most commonly affected areas were the head, followed by extremities, thorax, and abdomen, similar to results reported from Israel and Ireland [17,22]. Conversely, the incidence of chest, abdomen, spinal and pelvic, upper limb, and vascular injuries was gunshot injury patients compared to those injured by explosions. Similar findings were observed in a study conducted by Arslan et al. [23] in Somalia, which reported that explosive injuries predominantly affected the head, brain, and lower extremities, while the chest, abdomen, and pelvis were the most common injury sites for firearm wounds; however, injuries to all other body parts occurred more frequently in explosions.

The present study highlights a concerning trend in injury patterns, with intracranial hemorrhage, bowel injury, tibia fractures, and hemothorax being the most prevalent, particularly in explosion-related incidents, although intracranial hemorrhage is more common in gunshot wounds. A terrorist bomb attack in Madrid, Spain, led to a devastating situation, with reports indicating that the most frequent patterns of injuries sustained by the victims included rib fractures, long bone fractures, skull base fractures, and liver injuries [24]. A retrospective analysis of prospectively collected data from 181 patients with abdominal trauma admitted to Hadassah Hospital in Jerusalem, Israel, between October 2000 and December 2005 revealed that injuries to the small intestine and large intestine were the most prevalent intra-abdominal injuries following terror-related blast incidents [25]. Research conducted by Mohamed et al. [18] at a tertiary care hospital in Somalia found that fractures of the femur, tibia, and humerus were the most common types of limb injuries, especially in cases related to explosions.

In the current study, nearly half of the patients were admitted to medical wards, with a higher percentage of gunshot injury patients compared to explosion injury patients. A portion of the patients was admitted to the Intensive Care Unit (ICU) at similar rates for both groups, while some were taken directly to the operating theater, with slightly more explosion injury patients than gunshot injury patients. A study conducted in Israel found that ICU admission rates were approximately 22.8 %, with explosive injuries being slightly more common than those resulting from gunshot injuries [17]. Similar investigations from Somalia revealed that ICU admission rates were around 24 % and 10.2 %, with explosive injuries being the predominant type of injury [18,23]. Additionally, a study conducted in Israel demonstrated that a significant proportion of patients (51 %) underwent a surgical procedure, 58 % in the GSW group and 46 % in the explosion group [17].

By analyzing patient mortality, it's crucial to note that the data from this study do not include individuals who died at the scene or those who arrived at the hospital already deceased. However, 29 patients (12.9 %) passed away during their hospital stay, comparable to two retrospective studies reporting inpatient death rates of 6.3 % and 11.6 % ([17,23]).

The World Health Organization (WHO) defines MCIs as situations in which a greater number of patients than can be efficiently handled at once by local resources and standard operating procedures [1]. To respond to these situations effectively, extra resources, creativity, help, and unique emergency plans are usually required. Physicians may also need to obtain specialist knowledge in some fields, such as trauma emergency care, critical care medicine, emergency medicine, and trauma victim psychosocial support [26,17]. Physicians will be better equipped to handle the special challenges presented by MCI and treat affected patients promptly and appropriately, thanks to this expansion of their knowledge and skill set.

### Strengths and limitations

The study has several strengths and limitations. A notable strength is that the study addresses a significant public health concern, providing valuable insights that can inform emergency response strategies and resource allocation. Moreover, it allows for an in-depth analysis of injury patterns, demographic information, and treatment outcomes related to MCI. Additionally, the comparison between explosion-related injuries and gunshot wounds provides valuable information for targeted interventions. However, the study is not without limitations. It is a retrospective nature. The categorization of injuries may not account for all subtypes or complications, particularly in cases with multiple injuries. Excluding patients dead upon arrival is an other limitation, as their unregistered data could have been valuable. Furthermore, findings may not be generalizable to all contexts, as the study focused on a specific geographic region and patient population.

### Conclusion and recommendations

Gunshot and explosive injuries are the primary types of terror-related trauma in Somalia since the collapse of the central government. These injuries differ in severity, distribution, and outcomes, with younger males being the most affected. Explosion-related injuries are more prevalent and associated with higher mortality, particularly in the head and extremities. Additionally, the combination of inadequate training, limited resources, poor infrastructure, and low public awareness hinders prehospital care in Somalia. Based on these findings, emergency response protocols and EMS should be established. Healthcare providers require training, and preventive measures in high-risk areas can help reduce the severity of injuries and improve the outcome. Ongoing research is crucial for understanding the long-term outcomes and rehabilitation needs of these patients, ensuring they receive comprehensive care after discharge.

## Dissemination of results

The results of this study was presented to the government, as well as to local and international NGOs, to inform decision-making and improve resource allocation. One effective strategy involved submitting the findings to the African Journal of Emergency Medicine, to target a scholarly audience.

## Author contributions

HHM: Project administration, methodology, and writing- original draft. HAAA: Data curation and Validation. ST and MA: Data analysis, visualization, and editing. MFYM: Writing, critically revising, and supervision. All authors gave final approval of the version to be published; have agreed on the journal to which the article has been submitted; and agree to be accountable for all aspects of the work.

## Availability of data and materials

The datasets used and/or analyzed during the current study are available from the corresponding author (Mohamed Farah Yusuf Mohamud: m.qadar59@gmail.com) upon reasonable request.

## Funding

We declare that we have no any funding source.

## Declaration of competing interest

We declare that there is no conflict of interest associated with this publication and also there has been no significant financial support for his work that could have influenced its outcome.
